# Online Compensation of Phase Delay Error Based on P-F Characteristic for MEMS Vibratory Gyroscopes

**DOI:** 10.3390/mi13050647

**Published:** 2022-04-19

**Authors:** Xuewen Liu, Zhengcheng Qin, Hongsheng Li

**Affiliations:** 1 School of Instrument Science and Engineering, Southeast University, Nanjing 210096, China; 230208842@seu.edu.cn (X.L.); 230189281@seu.edu.cn (Z.Q.); 2 Key Laboratory of Micro-Inertial Instruments and Advanced Navigation Technology, Ministry of Education, Nanjing 210096, China

**Keywords:** MEMS gyroscopes, P-F characteristic, phase delay, online compensation

## Abstract

In this paper, an online compensation method of phase delay error based on a Phase-Frequency (P-F) characteristic has been proposed for MEMS Coriolis Vibratory Gyroscopes (CVGs). At first, the influences of phase delay were investigated in the drive and sense mode. The frequency response was acquired in the digital control system by collecting the demodulation value of drive displacement, which verified the existence and influence of the phase delay. In addition, based on the P-F characteristic, that is, when the phase shift of the nonresonant drive force through the resonator is almost 0° or 180°, the phase delay of the gyroscope is measured online by injecting a nonresonant reference signal into the drive-mode dynamics. After that, the phase delay is self-corrected by adjusting the demodulation phase angle without affecting the normal operation of the gyroscopes. The approach was validated with an MEMS dual-mass vibratory gyroscope under double-loop force-to-rebalance (in-phase FTR and quadrature FTR) closed-loop detection mode and implemented with FPGA. The measurement results showed that this scheme can detect and compensate phase delay to effectively eliminate the effect of the quadrature error. This technique reduces the zero rate output (ZRO) from −0.71°/s to −0.21°/s and bias stability (BS) from 23.30°/h to 4.49°/h, respectively. The temperature sensitivity of bias output from −20 °C to 40 °C has reached 0.003 °/s/°C.

## 1. Introduction

Microelectromechanical system (MEMS) Coriolis Vibratory Gyroscopes have been an increasingly significant inertial sensor due to their small size, light weight, low cost and high-density integration in recent years [1]. They have been employed widely in many applications, including balancing and aiming in consumer electronics [2], self-localization in autonomous vehicles [3], rotation angle detection in industrial automatic robots [4], medical equipment [5], and aerospace navigation [6].

MEMS CVGs act as an angular sensor based on the Coriolis principle [7], energy transfer occurs between two orthogonal modes—the drive mode and the sense mode—when working in normal operating conditions [8]. For operation, the drive mode realizes the resonant frequency tracking by a phase-locked loop (PLL) and the drive amplitude stabilization by an automatic gain control (AGC) loop [9]; the sense mode obtains the information about the input angular rate. In general, the drive mode is ideally completely orthogonal to the sense mode [10]. However, imperfection in the fabrication of the gyroscope resonator, such as anisodamping and anisoelasticity, would inevitably result in the coupling of two modes, where the quadrature error is the major error source in the rate gyroscopes [11,12]. As the quadrature error derives from the coupling of drive-mode displacement to the sense-mode according to the definition [13], it is 90° out of phase with the wanted Coriolis signal. A direct method to remove quadrature error is to use synchronous demodulation [14]. It is remarkable that the phase delay is introduced by the entire circuit of signal processing, digital signal processing modules and digital/analog conversion in the practical operations [15]. On one hand, it will cause the drive-mode in a nonresonant state, on the other hand, it will lead to the coupling between the Coriolis signal and the quadrature signal [16], which degrades the accuracy and the stability of gyroscopes [17]. Furthermore, the total phase delay varies with the temperature [18], so the online detection and compensation of the phase delay is significant in improving the performance of MEMS gyroscopes.

In order to compensate for the phase error, direct phase measurement and online compensation between the large quadrature output and the demodulation reference was applied in the open-loop detection system to improve the zero-rate output (ZRO) stability [19], which requires the large quadrature output and the high phase resolution instruments. A real time circuit phase delay correction system was proposed in the double-loop FTR control system based on the amplitude-frequency characteristic of the drive mode [20], which adjusted the reference value of PLL by detecting the minimum excitation force amplitude. Similarly, the maximum quadrature signal was monitored to obtain the phase error in the open loop detection system [21]. The disadvantage of the above two methods is the amount of time needed to search for the extreme-point. A two-stage capacitor amplifier circuit as a front-end amplifier [22] was conducted in an MEMS Quadruple Mass Gyroscope to reduce the overall phase shift of the loop, which also validated that the demodulation phase has a dominant impact in zero bias drift. A modified double sideband extraction algorithm was presented based on the symmetry of double sideband modulation [23] to eliminate the disturbance of circuit phase delay in the drive mode and sense mode. Moreover, a BP Neural Network was applied to establish an accurate compensation model [24] of a demodulation phase angle by training samples of resonant frequency and quality factor, which requires a higher arithmetic capability.

In this work, the influence of phase delay when gyroscopes work in a double-loop FTR control closed-loop detection state is discussed; the force-rebalance detection method is adopted here due to the advantages of improving the stability and the measurement range [25,26]. An online compensation of phase delay based on the P-F characteristic is proposed to detect and compensate the phase error of the overall loop by adding a nonresonant driving signal in the drive mode. The self-correction was achieved by collecting the orthogonal demodulation value of the nonresonant frequency displacement and adjusting the initial phase of the demodulation reference signal, then the residual phase error can be eliminated when the demodulation value is zero. This method is easy to implement and has good compatibility, which can be realized in the most existing gyro control system without additional analog circuits and complex digit arithmetic modules. Furthermore, the digital quantity of phase error in the whole loop is accessed precisely and in real time under the normal operating conditions. The compensation system can accurately measure and correct the phase delay online to remarkably weaken the coupling of quadrature error on the zero bias output, which provides the stratagem of phase delay self-calibration in future research and development.

The rest of this paper is structured as follows. The concept and dynamic model of the MEMS vibratory gyroscopes are introduced in Section 2. Then, Section 3 presents the digital control system and the influence of the phase delay. An online compensation method for phase delay based on the P-F characteristic is proposed in Section 4. Experimental results are presented in Section 5 and Section 6 presents the conclusion.

## 2. Gyroscope Dynamic Model

The vibratory gyroscope can be modeled as a second-order mass-damper-spring system, the dynamical equations of the gyroscope resonator model are [27]:

(1) $$\left[\begin{array}{cc} m_{x} & 0\\ 0 & m_{y} \end{array}\right]\;\left[\begin{array}{c} \ddot{x}\\ \ddot{y} \end{array}\right]\;+\;\left[\begin{array}{cc} c_{x} & c_{yx}\\ c_{xy}\; & c_{y}\; \end{array}\right]\;\left[\begin{array}{c} \dot{x}\\ \dot{y} \end{array}\right]\;+\;\left[\begin{array}{cc} k_{x} & k_{yx}\\ k_{xy}\; & k_{y} \end{array}\right]\;\left[\begin{array}{c} x\\ y \end{array}\right]\;=\;\left[\begin{array}{cc} 0 & 2m_{y}\Omega_{z}\left(t\right)\\ -2m_{x}\Omega_{z}\left(t\right) & 0 \end{array}\right]\;\left[\begin{array}{c} \dot{x}\\ \dot{y} \end{array}\right]\;+\;\left[\begin{array}{c} f_{x}\\ f_{y} \end{array}\right],$$

where the $k_{x}$, $c_{x}$ and $m_{x}$ represent the spring, damper and mass in the *x*-direction, and the $k_{y}$, $c_{y}$ and $m_{y}$ represent the spring, damper and mass in the *y*-direction, respectively. The parameters $c_{xy}$ and $c_{yx}$ denote the nonproportional damping, and $k_{xy}$ and $k_{yx}$ the anisoelasticity. The $\Omega_{z}\left(t\right)$ is the input angular velocity along the *z* axis, and $f_{x}$ and $f_{y}$ are external forces exerted on each direction of the resonator.

In general, the displacement of the sense mode under the closed-loop detection can be ignorable as a result of the balancing by feedback force. Therefore, the transfer function of the drive mode is simplified as:

(2) $$H_{x}\left(s\right)=\frac{1/m_{x}}{s^{2}+\omega_{x}/Q_{x}s+\omega_{x}^{2}},$$

where $\omega_{x}=\sqrt{k_{x}/m_{x}}$ is the resonance frequency and $Q_{x}=m_{x}\omega_{x}/c_{x}$ is the quality factor. We assume that the excitation force of the drive mode is $f_{x}\left(t\right)=A_{d}\sin\left(\omega_{d}t\right)$, where $A_{d}$ and $\omega_{d}$ are the excitation amplitude and the drive force frequency, respectively. Substituting Equation (3) into Equation (2), we obtain the expression of displacement during a steady state.

(3) $$x\left(t\right)=A_{d}\left|G\left(j\omega_{d}\right)\right|\sin\left(\omega_{d}t+∠G\left(j\omega_{d}\right)\right),$$

(4) $$A_{g}=\left|G\left(j\omega_{d}\right)\right|=\frac{1/m_{x}}{\sqrt{{\left(\omega_{x}^{2}-\omega_{d}^{2}\right)}^{2}+\omega_{x}^{2}\omega_{d}^{2}/Q_{x}^{2}}},$$

(5) $$\varphi_{x}^{*}=∠G\left(j\omega_{d}\right)=\left\{\begin{array}{c} -\arctan\frac{\omega_{x}\omega_{d}}{Q_{x}\left(\omega_{x}^{2}-\omega_{d}^{2}\right)}\;\;\;\omega_{d}\leq \omega_{x}\\ -\left(180^{{}^{\circ}}-\arctan\frac{\omega_{x}\omega_{d}}{Q_{x}\left(\omega_{d}^{2}-\omega_{x}^{2}\right)}\right)\;\omega_{d}>\omega_{x} \end{array}\right.,$$

where $A_{g}$ is the amplitude gain of the vibration displacement, and $\varphi_{x}^{*}$ is the phase shift in the resonator. The resonator is in resonant state when the drive frequency $\omega_{d}$ tracks the resonance frequency $\omega_{x}$, whose vibration amplitude gain $A_{g}$ reaches the maximum point and phase delay $\varphi_{x}^{*}$ is just $-90^{{}^{\circ}}$, which is the ideal relationship between phase shift and amplitude in the resonant state.

## 3. Phase Delay Analysis and Influences

### 3.1. Digital Control System

**Figure 1:**
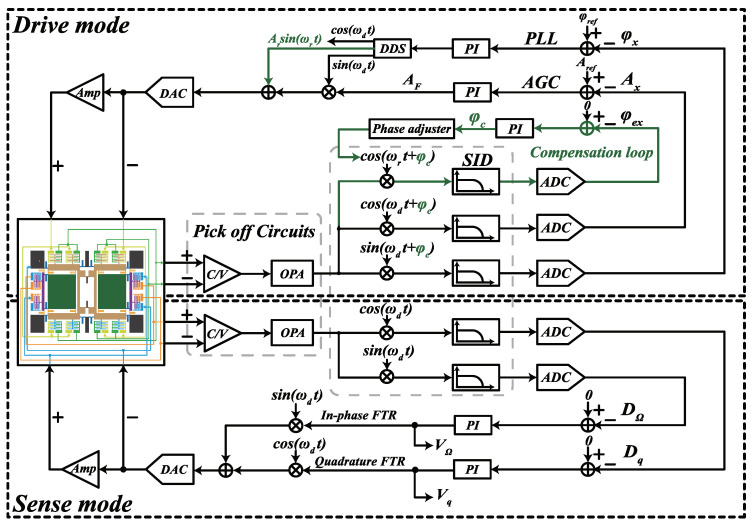
A block diagram of the digital control system.

The digital control system in this paper is depicted in Figure 1. The analog circuits consist of the pick-off circuit, the synchronous integral demodulator (SID) circuit, and the front-end amplifier. The pick-off circuit is composed of a C/V convert circuit and an operational amplifier (OPA), which is used to transfer the gyroscope comb capacitance displacement into the voltage signal and filter out the carrier signal, respectively. The SID circuit is applied to extract the in-phase and quadrature signal by each demodulation reference signal ($\sin\left(\omega_{d}t\right)$ and $\cos\left(\omega_{d}t\right)$) and low pass filter (LPF), and the front-end amplifiers are employed to generate the differential force signal for excitation and rebalance. A four channel ADC and two independent DACs are the bridges connecting analog circuits and digital systems.

In the drive mode, the PLL makes the driving frequency $\omega_{d}$ track resonant frequency $\omega_{x}$ and the AGC keeps the amplitude of the displacement $A_{x}$ at the reference amplitude $A_{ref}$. The demodulation reference and feedback force signals are originated by the Direct Digital Synthesis (DDS). The phase compensation loop is applied for adjusting the initial phase of the demodulation reference, and the details are introduced in Section 4. In the sense mode, the output signals are modulated by $\sin\left(\omega_{d}t\right)$ and $\cos\left(\omega_{d}t\right)$ to generate the two feedback forces $V_{\Omega }\sin\left(\omega_{d}t\right)$ and $V_{q}\cos\left(\omega_{d}t\right)$ by the PI controller under the double-loop FTR control, which keeps the sense mode relatively static. The output of the PI controller in the in-phase and quadrature loop are the angular velocity signal and quadrature error signal, respectively.

### 3.2. Phase Delay Influences under Double Closed Loop Detection

Sources of phase delay in the control system were demonstrated in detail in previous research [15,20,23]. Similarly, the complete loop phase delay $\varphi_{e}$ in the control system mentioned above can be divided into $\varphi_{DA}$, $\varphi_{Amp}$, $\varphi_{CV}$, $\varphi_{OPA}$, $\varphi_{code}$, which represent the phase delay of the DAC, the analog amplifier, CV convert circuit, OPA circuit, and the digital algorithm, respectively, that is, $\varphi_{e}=\varphi_{DA}+\varphi_{Amp}+\varphi_{CV}+\varphi_{OPA}+\varphi_{code}$. Now, considering the phase delay $\varphi_{e}$, the actual phase is as follows when in a resonant state.

(6) $$\varphi_{x}=\varphi_{x}^{*}+\varphi_{e}=-90^{{}^{\circ}}.$$

Equation (6) shows that the existence of phase delay directly causes the gyroscope to be in a nonresonant state since the phase shift $\varphi_{x}^{*}$ in the resonator is not $-90^{{}^{\circ}}$, which consequently affects the control accuracy. Besides, the research on the effect of phase delay in the drive mode is not mentioned in detail, so it is worth investigating the change of driving frequency $\omega_{d}$ and amplitude gain $A_{g}$ due to the phase delay, because the driving frequency $\omega_{d}$ is smaller than the resonant frequency $\omega_{x}$ in the actual operating system. Substituting Equation (6) into Equation (5), we can obtain the relationship of the driving frequency and the phase delay error.

(7) $$-90^{{}^{\circ}}-\varphi_{e}=-\arctan\frac{\omega_{x}\omega_{d}}{Q_{x}\left(\omega_{x}^{2}-\omega_{d}^{2}\right)};$$

the solution of the driving frequency can be acquired as:

(8) $$\omega_{d}=\frac{\omega_{x}-\sqrt{\omega_{x}^{2}+{\left(2Q_{x}\cot\varphi_{e}\right)}^{2}}}{2Q_{x}\cot\varphi_{e}}.$$

For directly evaluating the effect of the phase delay on the driving frequency, we could turn the target to the difference of the driving frequency $\omega_{d}$ and resonant frequency $\omega_{x}$, that is:

(9) $$\Delta \omega =\omega_{x}-\omega_{d}=\frac{\sqrt{\omega_{x}^{2}+{\left(2Q_{x}\cot\varphi_{e}\right)}^{2}}}{2Q_{x}\cot\varphi_{e}}.$$

Substituting hlEquation (9) into (4), we can acquire the expression of amplitude gain as follows:

(10) $$A_{g}=\frac{1/m_{x}}{\sqrt{{\left(\Delta \omega \right)}^{2}\times {\left(2\omega_{x}+\Delta \omega \right)}^{2}+\omega_{x}^{2}{\left(\omega_{x}-\Delta \omega \right)}^{2}/Q_{x}^{2}}}.$$

**Figure 2:**
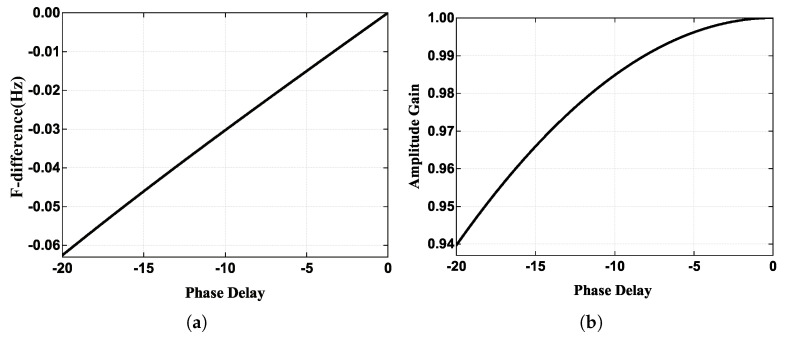
(**a**) The frequency difference between the driving frequency and resonant frequency. (**b**) The normalized value of amplitude gain.

Figure 2 shows the frequency difference Δω and the normalized value of amplitude gain as a function of the phase delay $\varphi_{e}$. It can be seen that the frequency difference is linearly related to the phase delay within the range from $-20^{{}^{\circ}}$ to $0^{{}^{\circ}}$, and the maximum deviation can reach 60 mHz, which cannot be neglected in the rate integrating gyroscope when tuning a two mode frequency. In addition, the phase delay would degrade the excitation efficiency in drive mode, which will cause the drive mode fail to work in severe cases. It quantified the effect of phase delay on the driving frequency and amplitude gain in driving mode for the first time.

**Figure 3:**
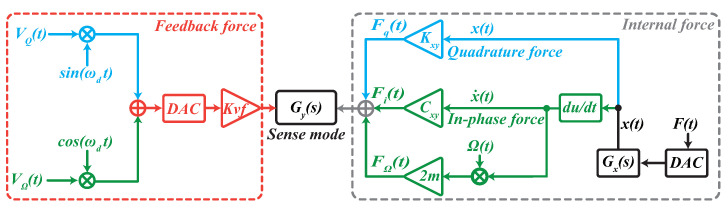
The force component analysis in sense mode.

For the sense mode, when ignoring other minor interference factors, such as driving force coupling and electrical detection cross-coupling, the force analysis in sense mode is shown in Figure 3.

The resultant internal force in sense mode includes Coriolis force $F_{\Omega }\left(t\right)$, in-phase damping coupling force $F_{i}\left(t\right)$ and quadrature error force $F_{q}\left(t\right)$, as suggested, respectively, in Equation (11).

(11) $$F_{\Omega }\left(t\right)=-2m_{y}\Omega \left(t\right)\dot{x}\left(t\right),\;F_{i}\left(t\right)=c_{xy}\dot{x}\left(t\right),\;F_{q}\left(t\right)=k_{xy}x\left(t\right).$$

Based on the phase delay error analysis above, the driving displacement can be adjusted to:

(12) $$x\left(t\right)=A_{x}\sin\left(\omega_{d}t+\varphi_{x}^{*}+\varphi_{DA}\right).$$

Substituting Equation (12) into Equation (11) acquires the resultant force $F_{y}\left(t\right)$ as follows:

(13) $$F_{y}\left(t\right)=\underset{F_{I}\left(t\right)}{\underset{︸}{\left(-2m_{y}\Omega \left(t\right)+c_{xy}\right)A_{x}\omega_{d}\cos\left(\omega_{d}t+\varphi_{x}^{*}+\varphi_{DA}\right)}}+\underset{F_{Q}\left(t\right)}{\underset{︸}{k_{xy}A_{x}\sin\left(\omega_{d}t+\varphi_{x}^{*}+\varphi_{DA}\right)}}.$$

In this paper, the double-loop FTR control holds the sense mode static by employing the feedback force generated by the DAC, which makes the residual voltage signal in the sense loop almost zero. Consequently, the demodulation of the Coriolis and quadrature signal is less insensitive to the change of phase shift $\varphi_{y}$ [28]. In addition, the relationship of the in-phase and quadrature feedback analog signals and sense mode forces is as follows according to the principle of FTR.

(14) $$F_{y}\left(t\right)+K_{vf}\left(V_{\Omega }\left(t\right)\sin\left(\omega_{d}t+\varphi_{DA}\right)+V_{Q}\left(t\right)\cos\left(\omega_{d}t+\varphi_{DA}\right)\right)=0,$$

where $V_{\Omega }\left(t\right)$ and $V_{Q}\left(t\right)$ illustrate the digital amplitude of in-phase and quadrature feedback signal digital amplitude respectively. Substituting Equation (13) into Equation (14) yields:

(15) $$\begin{array}{c} A_{\Omega }\left(t\right)\left[\cos\left(\omega_{d}t+\varphi_{DA}\right)\cos\left(\varphi_{x}^{*}\right)-\sin\left(\omega_{d}t+\varphi_{DA}\right)\sin\left(\varphi_{x}^{*}\right)\right]+\\ A_{Q}\left(t\right)\left[\sin\left(\omega_{d}t+\varphi_{DA}\right)\cos\left(\varphi_{x}^{*}\right)+\cos\left(\omega_{d}t+\varphi_{DA}\right)\sin\left(\varphi_{x}^{*}\right)\right]\\ =-K_{vf}\left\{V_{\Omega }\left(t\right)\sin\left(\omega_{d}t+\varphi_{DA}\right)+V_{Q}\left(t\right)\cos\left(\omega_{d}t+\varphi_{DA}\right))\right\}, \end{array}$$

where $K_{vf}$ is the transfer coefficient from voltage to force of gyroscope, $A_{\Omega }\left(t\right)$ and $A_{Q}\left(t\right)$ represent the amplitude of the in-phase and quadrature force, respectively.

(16) $$\left\{\begin{array}{c} A_{\Omega }\left(t\right)=\left(-2m_{y}\Omega \left(t\right)+c_{xy}\right)A_{x}\omega_{d}\\ A_{Q}\left(t\right)=k_{xy}A_{x}. \end{array}\right.$$

Combining Equations (15) and (16), then the Coriolis output equation is:

(17) $$V_{\Omega }\left(t\right)=\underset{Coriolis\;out}{\underset{︸}{[-2m_{y}A_{x}\omega_{d}\Omega \left(t\right)\sin\left(\varphi_{x}^{*}\right)}}-\underset{Quadrature\;error}{\underset{︸}{k_{xy}A_{x}\cos\left(\varphi_{x}^{*}\right)}}+\underset{In-phase\;error}{\underset{︸}{c_{xy}A_{x}\omega_{d}\sin\left(\varphi_{x}^{*})\right]}}/K_{vf}.$$

From the expression of the gyroscope output in Equation (17), it can be inferred as follows. First of all, the existence of phase error induces the $\varphi_{x}^{*}$ not to be $-90^{{}^{\circ}}$, which accordingly brings about the mixed coupling of Coriolis output and quadrature error, so the phase delay error will degenerate the bias stability of the gyroscope. Secondly, it was obvious that the Coriolis quantity $A_{\Omega }\left(t\right)$ is multiplied by a proportional coefficient $\sin(\varphi_{x}^{*})$, which consequently reduces the scale factor of the actual angular rate output. Furthermore, the effect of the damping coupling can be neglectable compared with the quadrature error [29]. As a result, the phase delay online compensation can effectively reduce the coupling error in Coriolis output, which is necessary for improving the performance of the gyroscope.

## 4. Phase Delay Compensation Methodology

### 4.1. Quantization of Phase Delay Error

**Figure 4:**
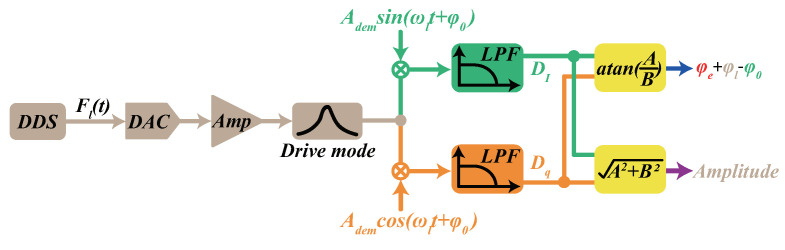
The sketch for quantization of the phase delay error.

The frequency response characterizations of the drive loop were acquired by collecting the digital data from the control system, as shown in Figure 4.

The input driving force $F_{l}\left(t\right)$ was generated by DDS and DAC with a fixed amplitude $F_{l}$ and a linearly increasing frequency $f_{l}$. Then, the quadrature demodulation digital quantity $D_{q}$ and the in-phase demodulation digital quantity $D_{I}$ were collected by the computer. Finally, the phase and amplitude values of the vibrating displacement were calculated by mathematical operations. We suppose that $F_{l}\left(t\right)=F_{l}\sin\left(\omega_{l}t\right)$. Combining the previous analysis mentioned, the expressions of the two demodulation outputs were established according to the demodulation principle.

(18) $$\begin{array}{cc} D_{I} & =\frac{1}{2}A_{l}\left[-\cos\left(2\omega_{l}+\varphi_{l}+\varphi_{e}+\varphi_{0}\right)+\cos\left(\varphi_{l}+\varphi_{e}-\varphi_{0}\right)\right]\\ \; & \overset{\Delta }{=}\frac{1}{2}K_{d}A_{l}\cos\left(\varphi_{l}+\varphi_{e}-\varphi_{0}\right), \end{array}$$

(19) $$\begin{array}{cc} D_{q} & =\frac{1}{2}A_{l}\left[\sin\left(2\omega_{r}+\varphi_{l}+\varphi_{e}+\varphi_{0}\right)+\sin\left(\varphi_{l}+\varphi_{e}-\varphi_{0}\right)\right]\\ \; & \overset{\Delta }{=}\frac{1}{2}K_{d}A_{l}\sin\left(\varphi_{l}+\varphi_{e}-\varphi_{0}\right), \end{array}$$

where the Δ denotes the LPF and ADC processes. $A_{l}$ and $\varphi_{l}$ represent the amplitude of driving displacement and the phase shift in the resonator respectively; $\varphi_{0}$ is the demodulation initial phase and $K_{d}$ is the gain during the whole driving loop. Lastly, the $\varphi_{e}$ represents the phase delay in the drive mode. So the phase and amplitude of the driving force through the whole driving loop is as follows:

(20) $$\varphi_{all}=at\;an\left(D_{q}/D_{I}\right)=\varphi_{l}+\varphi_{e}-\varphi_{0},$$

(21) $$A_{x}=\sqrt{D_{q}^{2}+D_{I}^{2}}=\frac{1}{2}K_{d}A_{l}.$$

**Figure 5:**
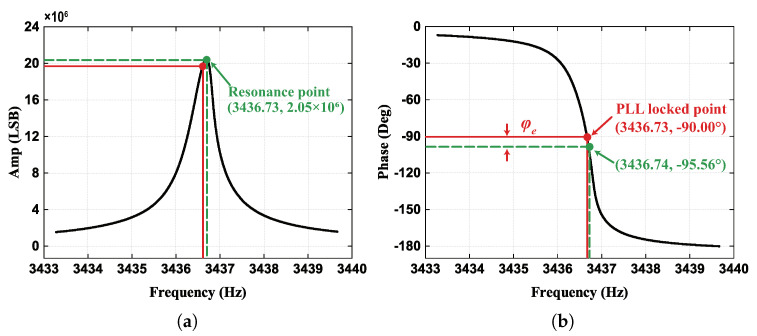
(**a**) The results of the phase-frequency characteristics. (**b**) The results of the amplitude-frequency characteristics. (The red solid line represent the actual condition, the green dashed line represent the resonant condition).

Figure 5 demonstrates the amplitude and phase frequency characteristics when we set the $\varphi_{0}$ to zero.

It is clear that the resonator is in a resonant state when $A_{x}$ reaches the maximum in Figure 5a, while the phase value $\varphi_{all}$ is not $-90^{{}^{\circ}}$ but an offset exists due to the phase delay error in Figure 5b from the calculation results. According to the control principle of PLL, the phase value is controlled at $-90^{{}^{\circ}}$, thus the existence of the phase delay error will lead the gyroscope resonator to not be in a resonant state. Besides, based on the fact that the phase $\varphi_{l}$ equals $-90^{{}^{\circ}}$ when the gyroscope is in resonant mode, the phase delay can be quantified by the phase frequency characteristic as:

(22) $$\varphi_{e}=\varphi_{all}-\varphi_{l}+\varphi_{0}=\varphi_{all}+90^{{}^{\circ}}-\varphi_{0}\left(\varphi_{0}=0^{{}^{\circ}}\right).$$

From the results in Figure 5b, we can obtain that the phase delay equals $-5.56^{{}^{\circ}}$, and the phase delay can be eliminated by adjusting the demodulation initial phase $\varphi_{0}$ from Equation (22). Another promising finding was that the phase shift $\varphi_{x}^{*}$ in nonresonant status is close to $0^{{}^{\circ}}$ or $-180^{{}^{\circ}}$ according to Equation (5). As a result, the phase delay can be characterized by the injected nonresonant force demodulation value. The detailed compensation scheme is demonstrated in the next section.

### 4.2. The Compensation Scheme for Phase Delay

**Figure 6:**
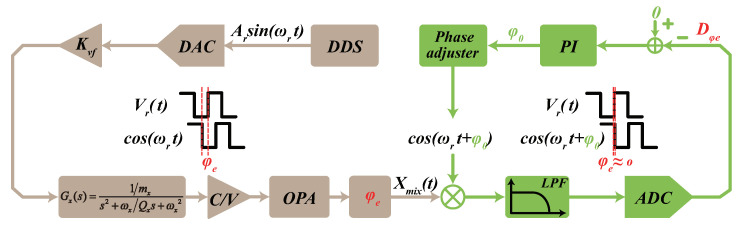
The functional block diagram of the compensation scheme.

As in the analysis above, an online compensation of the phase delay error is presented in double-loop FTR control. The compensation loop is shown in Figure 6. A nonresonant driving reference force $F_{r}\left(t\right)$ with a fixed amplitude $A_{r}$ and a fixed frequency $f_{r}$ was injected into the drive loop when the gyroscope is in normal operation. It is remarkable that the nonresonant frequency $f_{r}$ was always setting away from the driving frequency $f_{x}$, which can imitate the phase delay over the driving loop in the normal operation. In the meantime, the nonresonant frequency component could be separated by setting a suitable low-pass filter.

Firstly, the displacement of the drive mode becomes two frequency components.

(23) $$X_{mix}\left(t\right)=A_{d}\sin\left(\omega_{d}t+\varphi_{x}\right)+A_{r}\sin\left(\omega_{r}t+\varphi_{r}\right),$$

(24) $$\varphi_{x}=\varphi_{e}+\varphi_{x}^{*},$$

(25) $$\varphi_{r}=\varphi_{e}+\varphi_{r}^{*},$$

where $A_{d}$ and $A_{r}$ are the amplitude of the driving displacement and nonresonant displacement, $\varphi_{x}^{*}$ and $\varphi_{r}^{*}$ are the phase shift of driving force and nonresonant reference force through the resonator, respectively. After being demodulated by the demodulation signal $\cos\left(\omega_{r}t+\varphi_{0}\right)$, the output $D_{\varphi e}$ can be acquired according to Equation (19).

(26) $$\begin{array}{cc} D_{\varphi e} & =\frac{1}{2}K_{d}\underset{\omega_{r}+\omega_{d,r}}{\underset{︸}{[A_{d}\sin\left(\left(\omega_{d}+\omega_{r}\right)t+\varphi_{x}+\varphi_{0}\right)+A_{r}\sin\left(2\omega_{r}t+\varphi_{r}+\varphi_{0}\right)}}\\ \; & +\underset{\omega_{r}-\omega_{d}}{\underset{︸}{A_{d}\sin\left(\left(\omega_{r}-\omega_{d}\right)t+\varphi_{x}-\varphi_{0}\right)}}+\underset{\omega_{r}-\omega_{r}}{\underset{︸}{A_{r}\sin\left(\varphi_{r}-\varphi_{0}\right)}}]. \end{array}$$

It can be seen that the demodulation value is composed of four parts: $\omega_{r}+\omega_{d,r}$, $\omega_{r}-\omega_{d}$ and $\omega_{r}-\omega_{r}$; the DC component ($\omega_{r}-\omega_{r}$) represents the phase shift of the nonresonant force. By designing the suitable cut-off frequency of LPF and frequency difference $\omega_{r}-\omega_{d}$, the demodulation value is transferred as:

(27) $$D_{ref}=\frac{1}{2}K_{d}A_{r}\sin\left(\varphi_{r}-\varphi_{0}\right).$$

**Figure 7:**
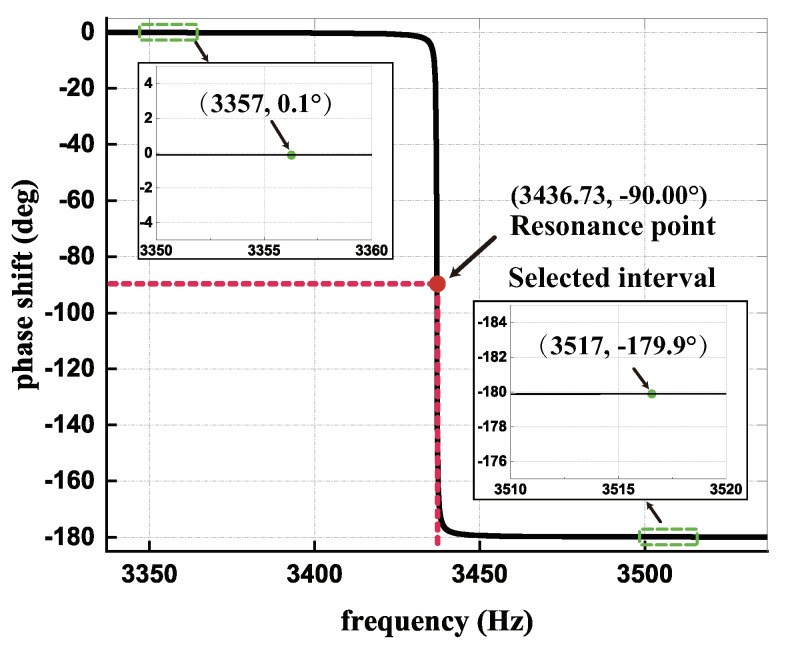
The P-F characteristic of the gyroscope resonator.

On the basis of the P-F characteristic of the gyroscope resonator from Equation (5), the phase shift through the resonator can be denoted as Figure 7.

It can be seen that the phase shift of nonresonant force through the resonator equals $0.1^{{}^{\circ}}$ or $-179.9^{{}^{\circ}}$ in the selected interval. Compared with the phase delay error measured before, the phase shift of nonresonant force can be approximated as:

(28) $$\varphi_{r}^{*}\approx 0^{{}^{\circ}}or-180^{{}^{\circ}}.$$

Moreover, the signal loop of nonresonant force is equivalent to the driving force ones; assuming that $\omega_{r}>\omega_{d}$ and combining Equations (25) and (28), Equation (27) can be modified as:

(29) $$D_{ref}=\frac{1}{2}K_{d}A_{r}\sin\left(-180^{{}^{\circ}}+\varphi_{e}-\varphi_{0}\right).$$

When the initial demodulation phase $\varphi_{0}$ offsets the phase delay $\varphi_{e}$, that is, $\varphi_{0}=\varphi_{e}$, Equation (29) can be simplified as:

(30) $$D_{ref}=\frac{1}{2}K_{d}A_{r}\sin\left(-180^{{}^{\circ}}\right)=0.$$

Accordingly, the phase delay can be removed by adjusting the initial demodulation phase when the demodulation value $D_{ref}$ is controlled to zero. Then, the compensation phase $\varphi_{0}$ is applied to the demodulation reference signal in drive mode, eventually correcting the misalignment between the displacement signal and demodulation signal caused by phase error in the drive mode.

**Figure 8:**
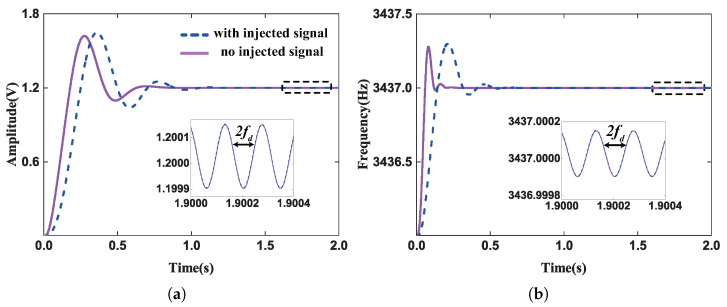
(**a**) The simulation for the amplitude of driving control signal. (**b**) The simulation for the frequency of driving control signal.

According to the principle of the compensation method, the feasibility of this method is that the nonresonant force does not affect the normal control in the drive mode. So the amplitude and frequency of the driving control signal are simulated before and after injecting the nonresonant reference force by Simulink. In order to keep the drive mode working in normal operation and not affect the bandwidth of the gyroscope, the nonresonant frequency was chosen to be 80 Hz higher than the driving mode natural frequency, and the cutoff frequency LPF in Figure 1 was lower than 80 Hz, which was applied to remove the frequency difference component. In this system, the cutoff frequency is designed to be 7 Hz. The simulation results are shown in Figure 8. Simulation results show that the injecting the reference signal would not affect the operation state of AGC and PLL owing to the low pass filter. The compensation loop and drive mode control loop were separated into their respective loops, and the double frequency component was far lower than the threshold of the control loop, so in the practical situation, this component can be ignored.

## 5. Experimental Results and Analysis

### 5.1. Experimental Setup

**Figure 9:**
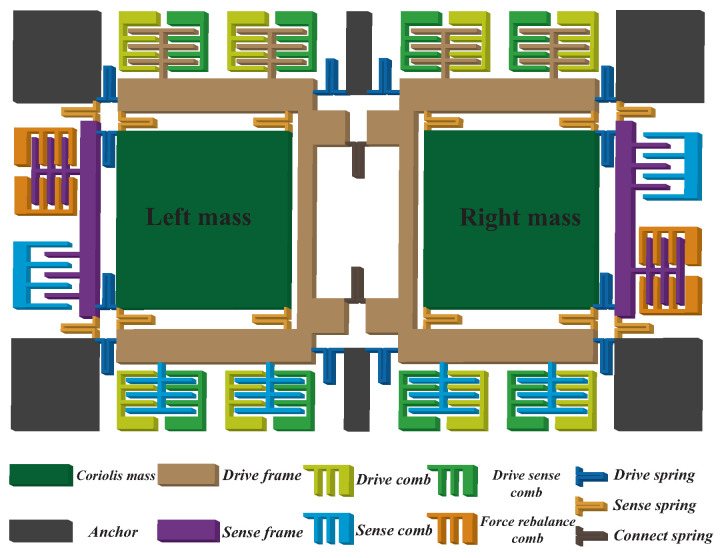
The solid figure of the gyroscope structure applied in this paper.

A vacuum-packaged dual-mass resonator gyroscope developing from the former structure [30] was applied in the experiment, whose structure is shown in Figure 9. The major parameters are listed in Table 1.

The main structure was composed of two symmetric single-mass gyroscopes, where the strings were used to connect the supporting frames and the effective mass, the anchors were applied to fix the resonator’s main structure to the glass substrate, the drive and force rebalance combs were used for exerting incentive and rebalance force, and drive sense and sense combs were used for detecting the displacements in drive and sense modes, respectively.

**Figure 10:**
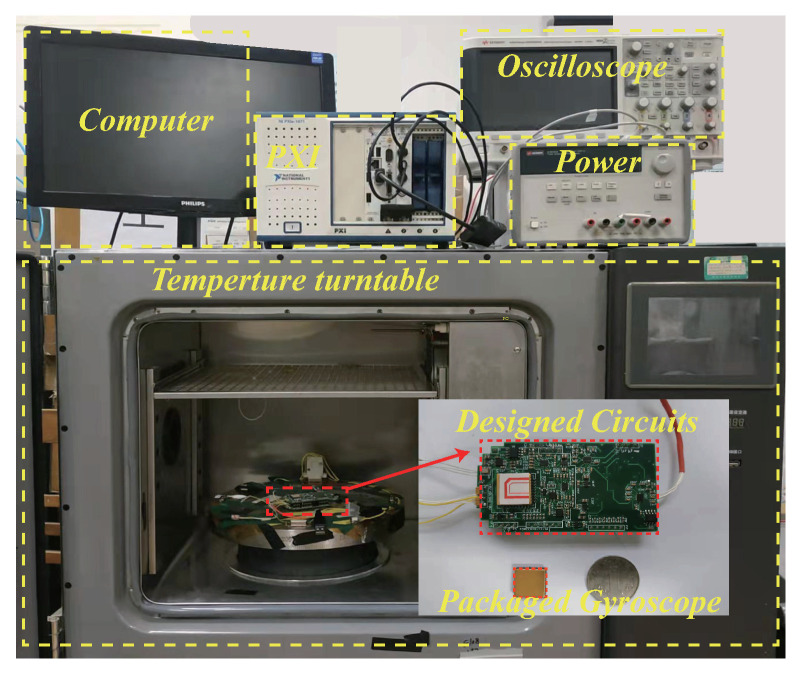
The picture of experimental platform.

Figure 10 shows the test environment and control circuits. The packaged gyroscope and circuit were fixed on the angle turntable wrapped in the incubator, the angle turntable was used to provide the angular rate and the incubator was applied to change different temperatures respectively. A direct current (DC) power supply was utilized to provide power and ground for the control system, and the Peripheral Component Interconnect (PXI) was used to measure the frequency spectrum of signals. The computer was applied to collect the test data through the USART by the LabVIEW at a 1 Hz sampling frequency.

### 5.2. Performance of Online Phase Delay Compensation

**Figure 11:**
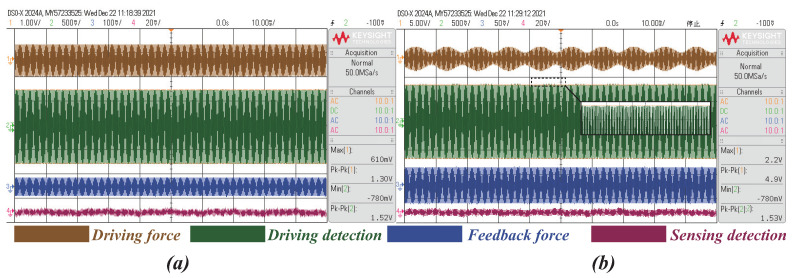
The oscillograms of the test signals. (**a**) No injecting reference signal. (**b**) With injecting reference signal.

**Figure 12:**
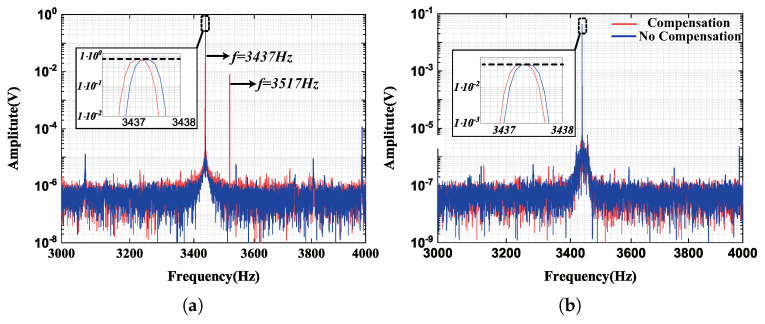
(**a**) The frequency spectrum of detection signal in drive mode. (**b**) The frequency spectrum of feedback force signal in sense mode.

Firstly, the signal waveforms in drive and sense modes are presented in Figure 11. From the oscillograms of the signals, we can observe that the injected reference signal would not influence the normal operation of the sense mode; the feedback force signal and detection signal in sense mode is still consistent after injecting the nonresonant frequency force, which indicates that the gyroscope is in the normal working state. For further verification, the change of the frequency spectrum of the drive mode detection signal and sense mode feedback force signal was investigated by PXI in Figure 12.

In Figure 12a, the driving displacement includes the resonance and nonresonant frequency component, which confirmed the detectability of the response of the reference signal. Besides, the amplitude of the resonant frequency component is equal before and after compensation, which shows that the nonresonant reference force would not affect the normal control of drive mode. In Figure 12b, the frequency spectrum of the feedback force signal in sense mode was matched identically in two situations, which demonstrated that the injecting signal would not affect the output of the gyroscope.

**Figure 13:**
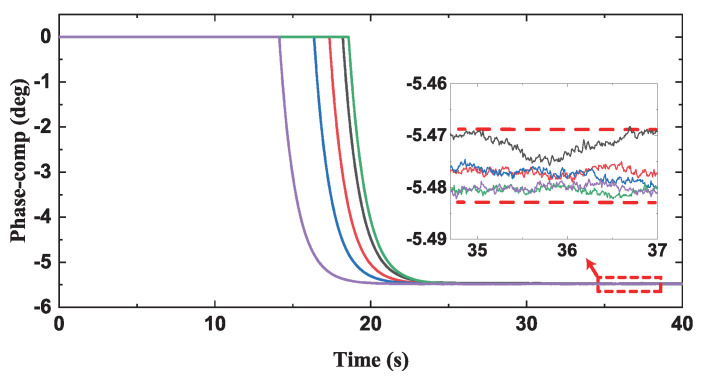
The five times measured results of the compensation phase.

The process of the compensation was tested five times in Figure 13. The phase compensation loop started working 20 s after the drive mode was in a stable state, and the compensation phase value tended to be stable after about 10 seconds and continued working. Moreover, the deviation of the stable phase value in the five measurements was within 0.1°. The difference between the final compensation phase value and the phase error in Figure 5 is within 0.2%, which proved the accuracy of the compensation scheme.

**Figure 14:**
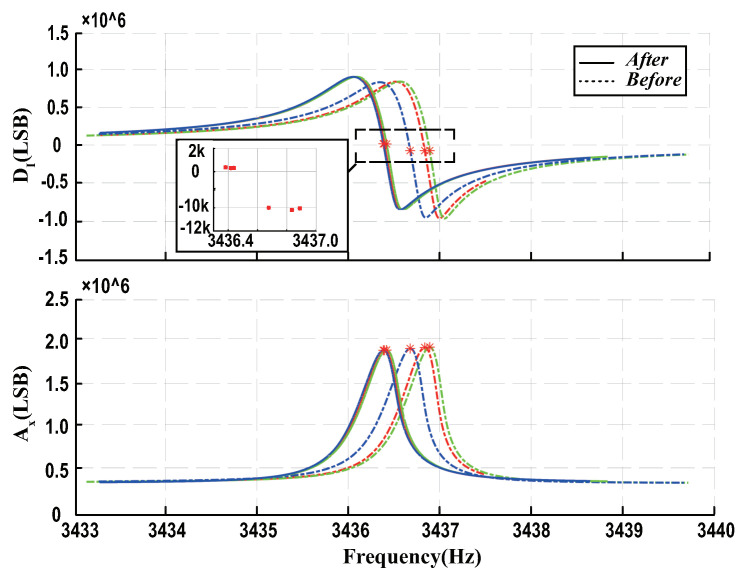
The results of the sweep frequency experiments before and after compensation.

In order to validate the accuracy and effectiveness of the proposed compensation method, the sweep frequency experiments were carried out on the digital control system as mentioned in Figure 4. The frequency response curves before and after compensation are shown in Figure 14, where $D_{I}$ and $A_{x}$ represent the digital quantity of the in-phase demodulation value and amplitude value. This can be easily seen by observing the residual in-phase demodulation value when the amplitude demodulation value reaches the maximum, and the residual offset introduced by the phase delay decreased by 98% from 100,000 LSB to 2000 LSB after compensation. The results demonstrated that the compensation scheme can remarkably eliminate the phase delay error, which makes the drive mode really work in the resonance state.

**Figure 15:**
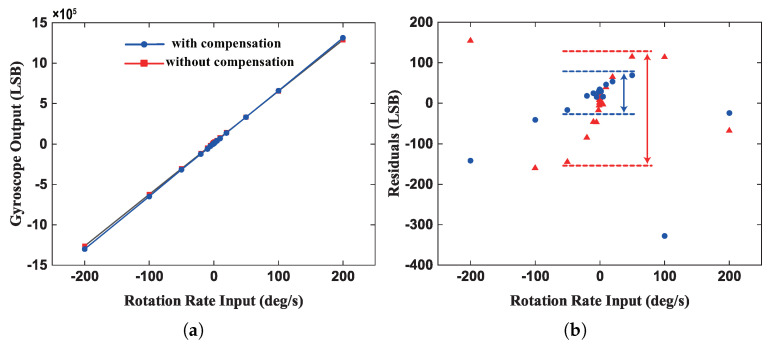
(**a**) Raw and compensated output of the gyroscope within $\;\pm 200^{{}^{\circ}}/s$. (**b**) The residual error of the scale factor in both cases.

The scale factor tests were arranged with input angular rates Ω of $\pm 0.1^{{}^{\circ}}/s$, $\pm 0.2^{{}^{\circ}}/s$, $\pm 0.5^{{}^{\circ}}/s$, $\pm 1^{{}^{\circ}}/s$, $\pm 2^{{}^{\circ}}/s$, $\pm 5^{{}^{\circ}}/s$, $\pm 10^{{}^{\circ}}/s$, $\pm 20^{{}^{\circ}}/s$, $\pm 50^{{}^{\circ}}/s$, $\pm 100^{{}^{\circ}}/s$, $\pm 200^{{}^{\circ}}/s$. The output of the gyroscope and the residual value in both cases are shown in Figure 15. From the experimental results, the compensated scale factor was increased from 6388 LSB/°/s to 6537 LSB/°/s, which reduces the leakage of Coriolis components. Besides, due to the suppression of the couple of the quadrature error, the nonlinearity of the scale factor reduced from 48.53 ppm to 27.41 ppm within $\pm 50^{{}^{\circ}}/s$, which improved the accuracy of the gyroscope output. However, the nonlinearity of the scale factor went from 62.80 ppm to 125.63 ppm within $\pm 200^{{}^{\circ}}/s$ since the nonresonant reference signal would affect the output of the gyroscope as the input angular velocity increasing, which indicated that the LPF would not completely filter out the reference frequency component. Therefore, this compensation scheme would be more suitable for a low-speed rotation scenario, such as North finder [31].

**Figure 16:**
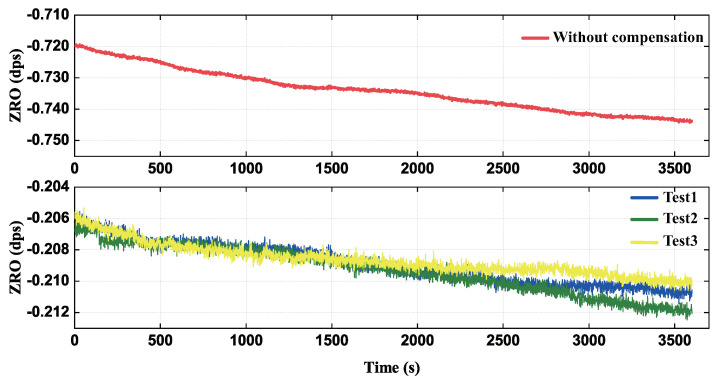
The ZRO of gyroscope at room temperature before and after compensation.

The ZRO of the gyroscope was recorded for 60mins by LabVIEW at room temperature, shown in Figure 16. The calculation results suggested that the bias stability (BS) and ZRO value are 23.301°/s and –0.717°/s before compensation, while the average of three tests of BS and ZB are 5.195°/h and –0.210°/s after compensation, which are improved by 3.41 times and 5.20 times, respectively. From the experimental data, the raw ZRO of gyroscopes was drifting because of a couple of quadrature errors, and the compensated ZRO were substantially reduced in both amplitude and drift values. In addition, the compensation data showed a great repetition in three tests. Our results demonstrated that the compensation can effectively offset the phase delay error and mitigate the leakage of quadrature error to the ZRO, which obtained the improvement in bias stability.

**Figure 17:**
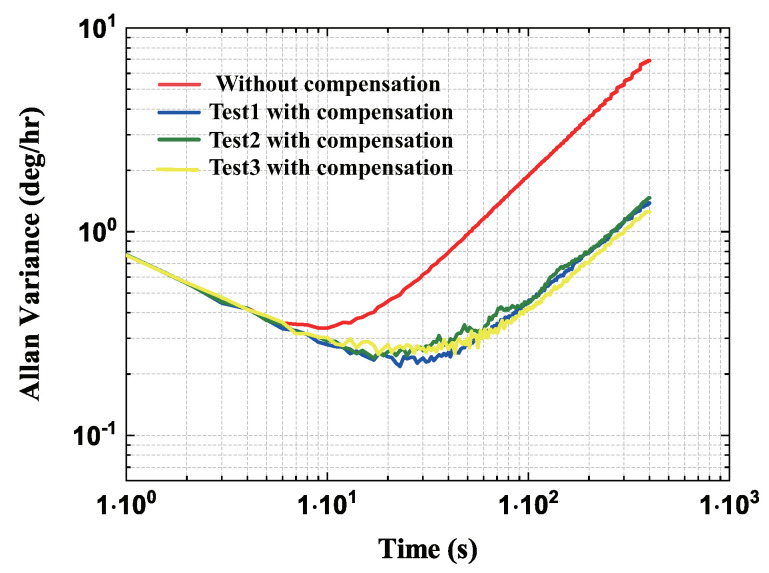
The Allan variance curve of ZRO before and after compensation.

Additionally, the Allan deviation curve of the ZRO for the two cases above were plotted in Figure 17. Results showed that the bias instability (BI) was enhanced from $0.338^{{}^{\circ}}/h$ to $0.236^{{}^{\circ}}/h$, but the angular random walk (ARW) was slightly worsening from $0.766^{{}^{\circ}}/h/\sqrt{\mathrm{hz}}$ to $0.769^{{}^{\circ}}/h/\sqrt{\mathrm{hz}}$ because of the injected reference signal. The results showed that the injected reference signal method would introduce additional noise, so the amplitude and frequency of the reference signal should be seriously considered in this method. In this paper, the deterioration of the ARW is acceptable compared with the improvement of the bias stability.

**Figure 18:**
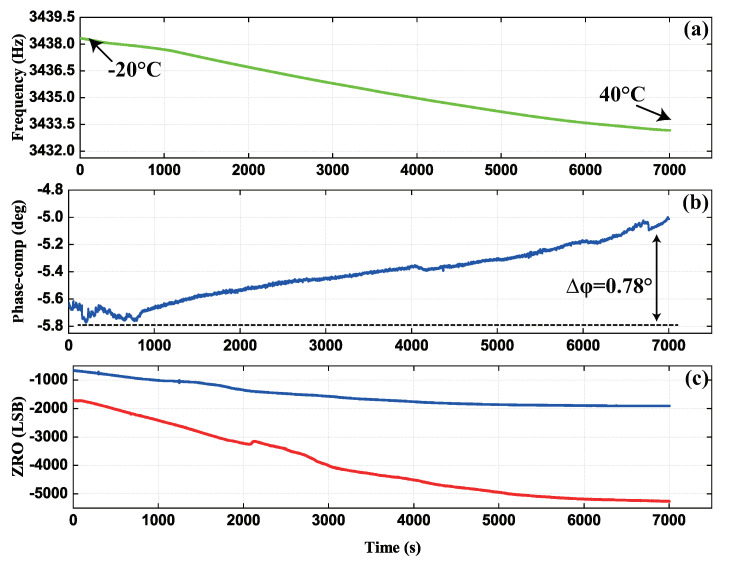
The ZRO of gyroscope at room temperature before and after compensation. (**a**) The driving frequency. (**b**) The variation of the compensation phase. (**c**) The ZRO before and after compensation.

Eventually, the gyroscope and circuits system was placed in the incubator to test the change of the compensation phase and the validity of the compensation scheme with the temperature range from −20 °C to 40 °C. As shown in Figure 18, the driving frequency was collected online to indicate the change of the interior temperature in the gyroscope control system. The maximum variation of the compensation phase is approximately 0.78° as the temperature changes from −20 °C to 40 °C. The variable quantity showed that the phase delay is sensitive to the temperature and online compensation is very necessary. Furthermore, it is significant to find that the variation of ZRO was reduced from 0.009°/s/°C to 0.003°/s/°C after compensation in the full temperature range. Although It also drifted due to the in-phase dampling error, the temperature sensitivity of the ZRO was decreased by three times with better temperature robustness. For one thing, the results confirmed the effect of the compensation program in eliminating the mixture of the quadrature error and depressing the temperature drift of ZRO, which also illustrates that the compensation method could track the fluctuation of the phase delay error online, despite the change of the temperature. It is also important for this method to relieve the influence of environmental temperature on the stability of the gyroscope.

## 6. Conclusions

We presented an online compensation for the phase delay error by injecting a known nonresonant driving signal as the reference signal to correct the misalignment between the driving displacement and the demodulation signal. The effect of phase delay on drive and sense mode was investigated and discussed by observing the digital data in the control system. A series of experiments were performed on the CVG by the described digital control system; our data indicated that the proposed compensation method can improve the ZRO and BS by 3.41 and 5.20 times to −0.21°/s and 4.49°/h respectively and achieve the performance of ARW and BI to 0.77°/h/$\sqrt{\mathrm{hz}}$ and 0.236°/h. This compensation methodology can effectively detect and eliminate the phase delay error for improving the stability of the MEMS gyroscopes. Future work will focus on the improvement of the performance of the LPF in our control circuits for better filtering out of the reference frequency component. Although this method would currently be applied to low-speed environments, we believe that this paper may provide a potential strategy for reducing the phase delay error in most applications.

## Figures and Tables

**Table 1:** Major parameters of the gyroscope.

Specification	Value	Units
Drive mode natural frequency $\omega_{x}$	3436.88Hz×2π	rad/s
Sense mode natural Frequency $\omega_{y}$	3457.12Hz×2π	rad/s
Quality factor of drive mode $Q_{x}$	8129.82	
Quality factor of sense mode $Q_{y}$	1262.92	
Mode Mass m	$6.70\times 10^{-7}$	kg
Drive mode vibration amplitude $A_{x}$	5.00	μm

## Data Availability

Not applicable.
